# Weight Fluctuation and Diet Concern Negatively Affect Food-Related Life Satisfaction in Chilean Male and Female Adolescents

**DOI:** 10.3389/fpsyg.2018.01013

**Published:** 2018-06-28

**Authors:** Berta Schnettler, Edgardo Miranda-Zapata, Klaus G. Grunert, Germán Lobos, Marianela Denegri, Clementina Hueche

**Affiliations:** ^1^Facultad de Ciencias Agropecuarias y Forestales, Universidad de La Frontera, Temuco, Chile; ^2^Escuela de Economía, Universidad Católica de Santiago de Guayaquil, Guayaquil, Ecuador; ^3^Centro de Excelencia en Psicología Económica y del Consumo, Núcleo Científico Tecnológico en Ciencias Sociales, Universidad de La Frontera, Temuco, Chile; ^4^Laboratorio de Investigación en Ciencias Sociales Aplicadas, Núcleo Científico Tecnológico en Ciencias Sociales, Universidad de La Frontera, Temuco, Chile; ^5^MAPP Centre, Aarhus University, Aarhus, Denmark; ^6^Programa de Investigación de Excelencia Interdisciplinaria en Envejecimiento Saludable, Facultad de Economía y Negocios, Universidad de Talca, Talca, Chile; ^7^Facultad de Educación, Ciencias Sociales y Humanidades, Universidad de La Frontera, Temuco, Chile

**Keywords:** dietary restriction, subjective well-being, adolescents, gender, invariance, structural equation modeling, moderating role

## Abstract

Problematic eating behaviors and obesity are frequent problems encountered during adolescence that may potentially result in psychological, social and physical consequences that may interfere with adolescent development and well-being. The present study evaluates the relationship between satisfaction with food-related life and satisfaction with family life, and their relationship with life satisfaction in male and female adolescents. We explored the relationships between both subscales of the Revised Restraint Scale (RRS), Diet Concern (DC) and Weight Fluctuation (WF) and adolescent life satisfaction as well as satisfaction with food-related life and family life. We also explored the moderating role of socioeconomic status (SES). A questionnaire was applied to a non-probabilistic sample of 470 adolescents (mean age 13.2 years, 52.3% female) in Chile, including the RRS, Satisfaction with Life Scale (SWLS), Satisfaction with Food-related Life (SWL-Food) scale and Satisfaction with Family Life (SWL-Family) scale. Using structural equation modeling, we found that adolescent life satisfaction was related to satisfaction with family life and food-related life in both genders. In male adolescents, a negative relationship was identified between WF and food-related life satisfaction. In contrast, a negative relationship was identified in female adolescents between DC and food-related life satisfaction. DC and WF were not directly related to life satisfaction or to satisfaction with family life in either gender. SES was found to moderate the relationship between food-related life satisfaction and life satisfaction and the relationship between WF and food-related life satisfaction in female adolescents. These findings suggest that reducing DC in female adolescents and reducing WF in male adolescents and female adolescents from higher SES may improve their food-related life satisfaction.

## Introduction

Problematic eating behaviors (such as overeating and restrained eating) and obesity are frequently experienced during adolescence (Loth et al., 2014; Walther and Hilbert, 2016) and can often persist into adulthood (Pearson et al., 2017). Dietary restraint is related to weight gain or increased body mass index (BMI) in adolescents of both genders (Snoek et al., 2008; Rhee et al., 2010; Forrester-Knauss et al., 2012) and has been identified as a risk factor in the development of eating disorders such as anorexia nervosa and bulimia nervosa, among others (Forrester-Knauss et al., 2012). Dietary restraint can also have negative consequences on adolescent psychological well-being or health (Proctor et al., 2009; Forrester-Knauss et al., 2012). Considering that adolescence is the period where individuals are most vulnerable to developing eating disorders (Cuesta-Zamora et al., 2018), this study focused on dietary restraint in adolescents of both genders and in the relationships between dietary restraint and adolescent life satisfaction and well-being in two life domains: food and family.

Recent studies have suggested that satisfaction within the food and family domains are positively correlated with overall life satisfaction, both in emerging adults (Schnettler et al., 2017c) and in adolescents (Schnettler et al., 2017b). Based on the bottom-up theoretical approach to life satisfaction, which implies that life satisfaction depends on the level of satisfaction a person feels in regard to different life domains (Brief et al., 1993), and the spillover model that proposes that domain satisfactions are positively related (Wu, 2009), the first objective of this study was to assess the joint relationship of the food and family domains with overall life satisfaction in adolescents from both genders. Based on the results previously obtained by Schnettler et al. (2017c) in a sample of emerging adults, we expected to confirm the following relationships in male and female adolescents:

H1: Satisfaction with food-related life is positively related to overall adolescent life satisfaction.
H2: Satisfaction with family life is positively related to overall adolescent life satisfaction.
H3: Satisfaction with food-related life is positively related to satisfaction with family life in adolescents.

Dietary restraint behavior refers to conscious attempts to reduce food intake in order to control body weight (Herman and Polivy, 1980; van Strien et al., 2012). However, this behavior is not clearly associated with reduced body weight. While some studies suggest that restraint may be a useful strategy in controlling body weight (van Strien et al., 2012; Girz et al., 2013; Schaumberg et al., 2016), others report that excessive restriction may have a counterproductive effect and may instead lead to overeating, binge eating, weight gain and the development of eating disorders in both genders (Herman and Polivy, 1980; Snoek et al., 2008; Masuda et al., 2015; Coffino et al., 2016; Walther and Hilbert, 2016). Lowe et al. (2013) concluded that, in most cases, restrained eating did not predict weight gain. Nevertheless, other authors have stressed that a higher BMI seems to not only be a result of restrained eating (Haines et al., 2007), but also a risk factor in the development of restrained eating (Forrester-Knauss et al., 2012). In fact, some authors reported that increased weight or BMI predicts dietary restraint in adolescents of both genders (Snoek et al., 2008; Forrester-Knauss et al., 2012), even though female adolescents usually have higher values of restrained eating than their male counterparts (Appleton and McGowan, 2006; Forrester-Knauss et al., 2012; Lai et al., 2013).

In this study, dietary restraint behavior was measured using the Revised Restraint Scale (RRS) (Herman and Mack, 1975; Herman and Polivy, 1980), the most widely used measure of restrained eating for the purpose of achieving or maintaining a desired weight. The RRS assesses “Diet Concern” (DC), the tendency of a person to restrain their food intake and their fear of weight gain, and “Weight Fluctuations” (WF), which register reported weight changes. Some authors have criticized the validity and internal consistency of the RRS in samples of overweight and obese individuals (Bohrer et al., 2015), while others have emphasized the prevalence of the DC subscale over WF in terms of its predictive capacity (van Strien et al., 2002, 2007). The WF subscale items may be problematic for assessing restraint in overweight individuals given that these individuals may obtain a high score on the RRS simply on the basis of large WFs, rather than an individual’s conscious decision to restrain their eating (van Strien et al., 2007). Likewise, it has been reported that the subscales of the RRS are not factorially simple (van Strien et al., 2002). In previous studies with females (van Strien et al., 2002) and undergraduate students of both genders (Schnettler et al., 2014) from Europe and South America, the RRS demonstrated the original two-factor structure (DC and WF). However, in these cases, it was necessary to omit items from both subscales in order to achieve an acceptable data fit. Similar results were reported by Mak and Lai (2012) in a mixed gender sample of adolescents in Hong Kong, though the results indicated a three-factor structure on the RRS. Although the 10-item version of the RSS has been validated in Spanish-speaking female undergraduate students (Silva, 2010) and adolescents (Silva and Urzúa-Morales, 2010), to our knowledge, its psychometric properties have not yet been assessed in male and female adolescents. Therefore, the present study addresses the factor structure of the RRS and its psychometric properties in both male and female adolescents. As part of assessing an instrument for use in the psychometric evaluation of adolescents, it is important to determine whether boys and girls ascribe the same meaning to the items of the RSS in terms of their underlying constructs (Moksnes et al., 2014). Therefore, measurement invariance of the RRS across gender was also tested.

In addition, to our knowledge, no studies exist that have assessed the relationships between both components of the RRS, life satisfaction, satisfaction with food-related life and satisfaction with family life. Therefore, the second aim of this study was to explore the relationships between DC and WF and adolescent life satisfaction as well as adolescent satisfaction with food-related life and family life.

Some studies have linked dietary restraint with poor psychological health (Ferreiro et al., 2014; Schnettler et al., 2014, 2017a; Tomba et al., 2014; Bentley et al., 2015; Mitchison et al., 2017) in male and female adolescents, although others have only reported this relationship in samples of female adolescents (Forrester-Knauss et al., 2012). Other researchers have studied the association between dietary restraint and life satisfaction in young people (Appleton and McGowan, 2006; Remick et al., 2009; Schnettler et al., 2014, 2016, 2017a; Orellana et al., 2016) with inconclusive results. While some authors reported that both variables are not associated in either gender (Appleton and McGowan, 2006), others have concluded that lower levels of life satisfaction were more related to WF than dietary concerns, regardless of gender (Schnettler et al., 2014). Likewise, dietary restraint was only found to be associated with reduced life satisfaction in females (Remick et al., 2009; Schnettler et al., 2017a), which aligns with the lack of association between these variables in male undergraduate students (Orellana et al., 2016). Similar results have been reported by Schnettler et al. (2014, 2017a) and Orellana et al. (2016) regarding the association between dietary restraint and levels of food-related life satisfaction. With this in mind, we explored the following relationships:

H4: Dietary concern is negatively related to adolescent life satisfaction.
H5: Weight fluctuation is negatively related to adolescent life satisfaction.
H6: Dietary concern is negatively related to adolescent satisfaction with food-related life.
H7: Weight fluctuation is negatively related to adolescent satisfaction with food-related life.

Some authors have reported that the family has a key role in shaping adolescent eating behavior (Loth et al., 2014; Towner et al., 2015; Schnettler et al., 2017b; Watts et al., 2017) and weight status (Berge et al., 2015; Towner et al., 2015; Tabbakh and Freeland-Graves, 2016). During family meals, parents use different food-related parenting practices to prevent excessive weight gain or obesity (Berge et al., 2015). However, some studies have reported that these practices may lead to a negative atmosphere during family meals (Salafia and Lemer, 2012), thereby affecting adolescent well-being (Salafia and Lemer, 2012; Skeer and Ballard, 2013; Utter et al., 2013; Schnettler et al., 2018a). Negative experiences during family meals may include discussions focused on food choices and weight, discussion of weight loss strategies (Berge et al., 2013) and verbal pressure from family members to be thin (Berge et al., 2013; Arroyo et al., 2017; Saunders and Frazier, 2017). Family meals have also been identified as moments of family unity that promote better family interaction and strengthen interpersonal relationships (Melbye et al., 2013; Ramalho et al., 2016; Salvy et al., 2017). Therefore, it is possible to expect that negative experiences related to diet and weight during family meals may negatively affect family relationships in adolescents. Given the above, we explored the following relationships:

H8: Dietary concern is negatively related to adolescent satisfaction with family life.
H9: Weight fluctuation is negatively related to adolescent satisfaction with family life.

Finally, given that the socioeconomic status (SES) category can influence some behaviors associated with life satisfaction, satisfaction with food-related life, satisfaction with family life and dietary restraint, we explored a moderating role of SES. Some authors have reported that adolescents and undergraduate students from lower SES are less likely to be satisfied with their life (Proctor et al., 2009; Schnettler et al., 2015a; Zou et al., 2018), food-related life (Schnettler et al., 2015a,b) and family life (Frasquilho et al., 2017; Gross-Manos, 2017). Regarding dietary restraint and SES, the availability of studies on this relationship is still scarce. However, some recent studies have suggested that adolescents from lower SES show less dietary restraint than their higher SES counterparts (Elgar et al., 2016; Godoy et al., 2018). Against this background, we tested the following hypothesis:

H10: SES moderates the relationship between satisfaction with food-related life and adolescent life satisfaction.
H11: SES moderates the relationship between satisfaction with family life and adolescent life satisfaction.
H12: SES moderates the relationship between dietary concern and adolescent life satisfaction.
H13: SES moderates the relationship between WF and adolescent life satisfaction.
H14: SES moderates the relationship between dietary concern and adolescent satisfaction with food-related life.
H15: SES moderates the relationship between WF and adolescent satisfaction with food-related life.
H16: SES moderates the relationship between dietary concern and adolescent satisfaction with family life.
H17: SES moderates the relationship between WF and adolescent satisfaction with family life.

## Materials and Methods

### Sample and Procedure

Non-probability sampling was used to recruit a sample of adolescents between 10 and 17 years of age from both dual-headed families and single-headed families in Temuco, Chile. In the case of dual-headed families, children from married mothers and unmarried cohabiting mothers were included, as a growing preference for cohabitation in lieu of legal marriage has recently been observed in Chile (Calvo et al., 2011). In the case of single parent families, such families were only included if the mother was the head of the household. Single parent families where the father was the head of the household were excluded as this is an uncommon scenario in Chile (CASEN, 2015).

**FIGURE 1:**
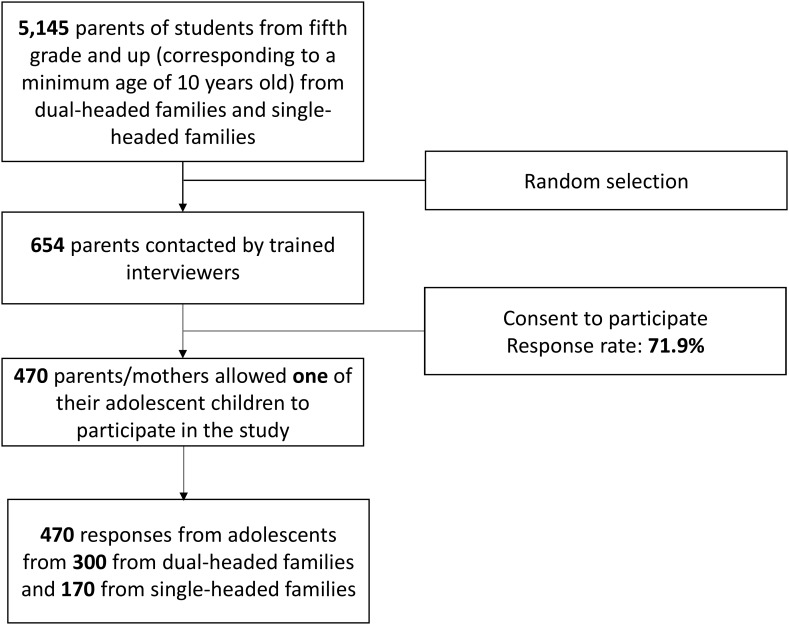
Flow chart of participant recruitment.

Participants were recruited from seven schools that serve socioeconomically diverse populations. Directors at each school signed authorization letters to allow research to be conducted with their students and provided a list containing 5,145 students from fifth grade upward (corresponding to a minimum age of 10 years) along with their parents’ telephone numbers. From the list of 5,145 parents, 654 were chosen using simple random sampling and contacted by trained interviewers, who explained the objectives of the study and the strictly confidential treatment of the information obtained. Then, they provided detailed information about the questionnaires and asked if he/she would allow one of their children between 10 and 17 years old to participate in the study. A total of 470 parents/mothers (300 from dual-headed families and 170 from single-headed families) allowed their child to participate, resulting in a response rate of 71.9%. Of the 654 parents/mothers contacted from the list, there were no single father families (see flow chart in **Figure 1**).

Interviews were conducted in the participants’ homes or schools, according to their preference. After all parents had signed written informed consent forms and adolescents had signed assent forms, the questionnaires were administered by a trained interviewer to the adolescent without his/her family members present. The anonymity of the respondents was ensured. The study was conducted between June and December 2016. The design of the study was approved by the Ethics Committee of Universidad de La Frontera (protocol number, 005/2016). A pilot test of the questionnaires was conducted with 20 adolescents following the same recruitment method. As the pilot test of the instrument was satisfactory, no changes were made to either the questionnaires or the interview procedure.

### Instruments

The questionnaire was composed of the following scales:

- *Revised Restraint Scale* (RRS; Herman and Mack, 1975; Herman and Polivy, 1980): the RRS is a 10-item scale that consists of two subscales: DC (e.g., “*Do you give too much time and thought to food?*”) and WF (e.g., “*What is the maximum amount of weight (in kilos) you have ever lost within 1 month?*”). Options for the responses are on a 4-point Likert scale for the DC subscale and a 5-point Likert Scale for the WF subscale. The scores provide a measure of chronic food restriction and range between 0 and 44. Higher scores indicate stricter dietary restraint (Lai et al., 2013). In this research study, the Spanish version of the RRS (Silva, 2010) was used, which has shown adequate levels of internal consistency for each subscale (DC = 0.68–0.78, WF = 0.71–0.79) in previous studies conducted in Chile with female adolescents and undergraduate students (Silva, 2010; Silva and Urzúa-Morales, 2010).
- *Satisfaction with Life Scale* (SWLS): SWLS (Diener et al., 1985) is a scale composed of five items grouped into a single dimension that is used to evaluate overall cognitive judgments about a person’s own life (e.g., *“In most ways my life is close to my ideal”*). The Spanish version of the SWLS (Schnettler et al., 2011) was used, which has shown adequate levels of internal consistency (Cronbach α = 0.79–0.91) in previous studies with undergraduate students and adolescents (Schnettler et al., 2013, 2014, 2015b, 2016, 2017a,b,c, 2018a,b). Respondents were asked to indicate their degree of agreement with each statement using a 6-point Likert scale (1: completely disagree; 6: completely agree). The scale showed good internal reliability (Cronbach α female adolescents = 0.922, Cronbach α male adolescents = 0.886). SWLS scores were obtained by summing up the scores from the five items.
- *Satisfaction with Food-related Life* (SWL-Food): SWL-Food (Grunert et al., 2007) is a scale consisting of five items grouped into a single dimension that is used to evaluate a person’s overall assessment of their food and eating habits (e.g., *“Food and meals are positive elements”*). The Spanish version of the SWL-Food (Schnettler et al., 2011) was used, which has shown adequate levels of internal consistency (Cronbach α = 0.76–0.90) in previous studies with undergraduate students and adolescents (Schnettler et al., 2013, 2014, 2015b, 2017a,b,c, 2018a,b). Respondents were asked to indicate their degree of agreement with each statement using a 6-point Likert scale (1: completely disagree; 6: completely agree). The scale showed good internal reliability (Cronbach α female adolescents = 0.914, Cronbach α male adolescents = 0.890). SWL-Food scores were obtained by summing up the scores from the five items.
- *Satisfaction with Family Life* (SWL-Family) scale. This scale, proposed by Zabriskie and McCormick (2003), is an adaptation of the SWLS (Diener et al., 1985) in which the words “family life” replaces the word “life” in each of the five original items of the SWLS. Family satisfaction can be defined as a conscious cognitive judgment of one’s family life based on the subjective criteria of each individual (Zabriskie and McCormick, 2003). The Spanish version of the SWL-Family (Schnettler et al., 2017c) was used, which has shown good levels of internal consistency (Cronbach α = 0.90–0.92) in previous studies with undergraduate students and adolescents (Schnettler et al., 2017b,c, 2018a,b). Respondents were asked to indicate their degree of agreement with each statement using a 6-point Likert scale (1: completely disagree; 6: completely agree). The scale showed good internal reliability (Cronbach α female adolescents = 0.935, Cronbach α male adolescents = 0.895). SWL-Family scores were obtained by summing up the scores from the five items.

The discriminant validity between the SWLS, the Satisfaction with Food-related life scale and the Satisfaction with Family Life scale was previously demonstrated in samples of undergraduate students and adolescents in Chile (Schnettler et al., 2017b,c). Therefore, although the different measures of life satisfaction share content in their questions, the aforementioned studies have shown that each scale measures a different construct.

Adolescents were asked to indicate their age, number of family members and family structure. In addition, they were asked to provide their approximate weight and height, which was used to calculate BMI (kg/m^2^). Education level and occupation of the head of the household were used to determine the SES of the family (Adimark, 2004).

### Statistical Analyses

Descriptive analyses were conducted using SPSS v.23 (all variables analyzed in the study are included in the Supplementary Data Sheet 1). Confirmatory factor analysis (CFA), multi-group analysis and structural equation model (SEM) were conducted using MPlus 7.11. Parameters of the measurement models (factor loadings) and structural models (path coefficients) were estimated using the Robust Maximum Likelihood (RML) estimator, which provides robust estimates in the presence of non-normality and the ordinal nature of Likert scales with five or more response categories (Rhemtulla et al., 2012; Finney and DiStefano, 2013).

First, to evaluate the psychometric properties of the RRS, a CFA was carried out using the 10 original items proposed by Herman and Polivy (1980). A factor loading lower than 0.4 was considered as a criterion for the elimination of an item (Stevens, 2009). The Omega coefficient was used to examine the reliability and internal consistency of the subscales (McDonald, 1970). The Chi-square (χ^2^), Tucker-Lewis Index (TLI) and Comparative Fit Index (CFI) were used to determine model fit of the data. If the model shows a statistically non-significant Chi-square result, then a good fit is reached. The TLI and CFI indicate a good fit with a value above 0.95, while 0.90 is considered a cut-off point for establishing an acceptable fit. In addition, the Root Mean Square Error of Approximation (RMSEA) was considered. The RMSEA is a poorness of fit measurement. A good fit is found when the value of the RMSEA is lower than 0.06, whereas an acceptable fit corresponds to a value lower than 0.08 (Hu and Bentler, 1999; Marsh et al., 2005).

Second, a multi-group analysis was developed to test measurement invariance of the RRS across genders. Configural invariance and measurement invariance (weak invariance, strong invariance and strict invariance) were verified (Dimitrov, 2010). In order to determine configural invariance, the same fit indices described for the CFA were used. In order to establish measurement invariance, a non-significant Delta from the Satorra–Bentler scaled Chi-square test (D_SBS χ^2^) was required (Satorra and Bentler, 2001).

Third, a SEM was used to test the proposed relationships between life satisfaction, satisfaction with food-related life, satisfaction with family life and between both sub-scales of the RRS and the aforementioned constructs. SEM was chosen for this analysis because it allows multiple relationships between latent variables to be visualized, including both the error of measurement and error of prediction in a single analysis (Ruiz et al., 2010). Considering that dieting concerns are different between male and female adolescents (Gallant et al., 2012; Lai et al., 2013; Ramos et al., 2013), hypotheses were tested separately by gender. To evaluate the goodness of fit of the models, the same fit indices described for the CFA were used.

Finally, in order to test the moderating role of SES, multi-group analyses were performed in order to establish the differences between the structural parameters (relationship between satisfaction with food-related life and life satisfaction, relationship between satisfaction with family life and life satisfaction, relationship between DC and life satisfaction, relationship between WF and life satisfaction, relationship between DC and satisfaction with food-related life, relationship between WF and satisfaction with food-related life, relationship between DC and satisfaction with family life, and relationship between WF and satisfaction with family life). Hypotheses were tested separately by gender. In order to create more heterogeneous groups, the multi-group analyses separately considered high and upper-middle and middle-middle statuses as “High SES” (37.0%) and lower-middle, low and very low statuses as “Low SES” (63%).

## Results

### Sample Description

A total of 470 adolescents aged between 10 and 17 years participated in the study. A total of 52.3% of the adolescents were female. **Table 1** shows the sociodemographic characteristics of the total sample and subsamples according to gender. There was a higher proportion of male adolescents living in dual-headed households than female adolescents (*P* ≤ 0.05). Subsamples did not significantly differ in the other socio-demographic characteristics examined (*P* > 0.1). **Table 1** also shows the composition of the total sample and both subsamples according to participant BMIs as per the criteria of the World Health Organization [WHO] (2007) and the Technical Norm of Nutritional Evaluation of children from 5 to 19 years of age developed by the Ministerio de Salud de Chile [MINSAL] (2016). Male and female adolescents did not differ in their distribution according to BMI (*p* > 0.1). Male and female adolescents did not differ in their DC, WF and RRS average scores (*p* > 0.1). Male adolescents had significantly higher average scores on the Satisfaction with Food-related Life, Satisfaction with Family Life and SWLSs than female adolescents (*p* ≤ 0.05).

**Table 1:** Socio-demographic characteristics, body mass index (BMI) and average scores on the satisfaction with food-related (SWL-Food), satisfaction with family life (SWL-Family) and Satisfaction with life (SWLS) scales, dietary concern (DC), weight fluctuation (WF) subscales and Revised Restraint Scale (RRS) of the sample.

Characteristic	Total (*n* = 470)	Female (*n* = 246)	Male (*n* = 224)	*P*-value
Age [Mean (*SD*)]^1^	13.3 (2.3)	13.3 (2.3)	13.2 (2.4)	0.553
Number of family members [Mean (*SD*)]^1^	4.1 (1.3)	4.0 (1.2)	4.2 (1.3)	0.228
Family structure^2^		
Dual-headed household	63.8	59.2	68.8	0.034
Single-headed household	36.2	40.7	31.2	
Socioeconomic status (%)^2^		
High and upper-middle	16.7	15.7	17.7	0.756
Middle–Middle	20.3	20.2	20.5	
Lower–Middle	33.1	35.5	30.5	
Low	23.6	23.1	24.1	
Very low	6.3	5.4	7.3	
BMI (%)^2^		
Undernourished (≤-2 SD)	6.8	5.7	8.0	0.894
Underweight (≤−1 to −1.9 SD)	13.4	13.8	12.9	
Normal range (+0.9 to −0.9 SD)	56.2	56.9	55.4	
Overweight (≥ +1 to +1.9 SD)	18.1	17.9	18.3	
Obesity (≥ +2 SD)	5.5	5.7	5.4	
SWL-Food [Mean (*SD*)]^1^	22.6 (6.3)	21.8 (6.7)	23.5 (5.6)	0.003
SWL-Family [Mean (*SD*)]^1^	24.1 (5.7)	23.4 (6.4)	24.8 (4.7)	0.007
SWLS [Mean (*SD*)]^1^	23.9 (5.6)	23.4 (6.1)	24.5 (4.8)	0.034
DC [Mean (*SD*)]^1^	10.5 (3.5)	10.7 (3.7)	10.2 (3.2)	0.130
WF [Mean (*SD*)]^1^	6.7 (2.1)	6.6 (2.1)	6.8 (2.3)	0.229
RSS [Mean (*SD*)]^1^	17.1 (5.1)	17.1 (5.2)	17.5 (4.8)	0.933

*^1^Independent sample t-test. ^2^Pearson Chi-Square Test.*

**Table 2** shows the relationship between the same variables and the adolescents BMI. Undernourished and underweight adolescents were significantly younger than normal weight, overweight and obese adolescents (*p* ≤ 0.001). BMI categories did not significantly differ in the other socio-demographic characteristics examined (*p* > 0.1). BMI categories also did not differ in their average scores on the satisfaction with food-related life, satisfaction with family life and life satisfaction scales (*p* > 0.1). Undernourished and underweight adolescents had DC average scores significantly lower than overweight and obese adolescents (*p* ≤ 0.05). Underweight adolescents had average WF scores that were significantly lower than other BMI categories (*p* ≤ 0.001). Undernourished and underweight adolescents had average RRS scores that were significantly lower than those of normal weight, overweight and obese adolescents (*p* ≤ 0.001).

**Table 2:** Socio-demographic characteristics and average scores on the SWL-Food, SWL-Family and SWLS scales, DC, WF subscales and RSS according to the body mass index (BMI) of the sample.

Characteristic	Undernourished (*n* = 32)	Underweight (*n* = 63)	Normal Range (*n* = 264)	Overweight (*n* = 85)	Obesity (*n* = 26)	*P*-value
Age [Mean (*SD*)]^1^	12.0 (2.0)b	12.2 (1.9)b	13.4 (2.3)a	13.6 (2.1)a	13.3 (2.4)a	<0.001
Number of family members [Mean (*SD*)]^1^	4.2 (1.6)	3.9 (1.2)	4.0 (1.3)	4.1 (1.0)	4.0 (0.9)	0.777
**Family structure (%)^2^**		
Dual-headed household	40.6	34.9	39.0	30.6	23.1	0.364
Single-headed household	59.4	65.1	61.0	69.4	76.9	
**Socioeconomic status (%)^2^**		
High and upper-middle	19.4	22.2	14.7	17.9	15.4	0.303
Middle–Middle	32.3	9.5	22.5	21.4	7.7	
Lower–Middle	19.4	39.7	34.1	27.4	42.3	
Low	22.6	19.0	22.5	28.6	30.8	
Very low	6.5	9.5	6.2	4.8	3.8	
SWL-Food [Mean (*SD*)]^1^	23.9 (6.2)	21.7 (6.9)	22.8 (6.1)	21.6 (6.1)	23.5 (6.0)	0.254
SWL-Family [Mean (*SD*)]^1^	24.9 (5.7)	24.1 (6.3)	24.1 (5.5)	23.4 (5.9)	24.3 (5.1)	0.758
SWLS [Mean (*SD*)]^1^	26.4 (3.9)	23.5 (7.2)	23.8 (5.2)	23.4 (5.4)	24.2 (5.4)	0.106
DC [Mean (*SD*)]^1^	9.2 (3.1)b	9.4 (3.1)b	10.5 (3.5)ab	11.1 (3.1)a	11.0 (3.6)a	0.007
WF [Mean (*SD*)]^1^	6.8 (2.2)a	5.6 (1.5)b	6.6 (2.2)a	7.1 (2.0)a	7.2 (2.6)a	0.001
RSS [Mean (*SD*)]^1^	15.4 (4.7)b	14.7 (3.8)b	17.1 (5.1)a	18.4 (4.6)a	19.1 (5.6)a	<0.001

*^1^One way analysis of variance. Different letters indicate statically significant differences according to Tukey Comparison test for homogenous variances. ^2^Pearson Chi-Square Test.*

### Psychometric Properties of the RRS

The RSS with 10 items showed a significant χ^2^ test. However, it is well established that χ^2^ test is sensitive to sample size (MacCallum et al., 2006), as was the case in this study. Therefore, considering the remaining indices of fit, the RSS showed an acceptable fit with the data from the sample of 470 adolescents of both genders (**Table 3**). However, item 7 showed a factor loading lower than 0.4, which is why it was removed. Upon the elimination of item 7, the goodness of fit indices varied but stayed within the acceptable range. On the other hand, the value of the Omega coefficient increased in the DC factor. Considering that this model (without item 7) is more parsimonious, it was used in subsequent analyses.

**Table 3:** Fit indices of the RRS confirmatory factor analysis (CFA).

Model	χ^2^	df	RMSEA	CFI	TLI	ωDC	ωWF
CFA original model	77.979	34	0.052	0.927	0.903	0.674	0.693
CFA model without item 7	66.590	26	0.058	0.928	0.900	0.681	0.693

χ*^2^, Chi square. df, Degree of freedom. RMSEA, Root Mean Square Error of Approximations. CFI, Comparative Fit Index. TLI, Tuker–Lewis Index.* ω*DC, Omega coefficient for the dietary concern (DC) subscale of the RRS.* ω*WF, Omega coefficient for the weight fluctuation (WF) subscale of the RRS.*

The multi-group analysis of the RSS without item 7 (**Table 4**) showed a significant χ^2^ test. However, considering the study sample size (MacCallum et al., 2006), the overall fit of the baseline model (without invariance) was acceptable, which established the configural invariance of the RSS across gender (**Table 4**). The non-significant Deltas from the Satorra–Bentler scaled Chi-square test (D_SBS χ^2^) indicated the achievement of strict invariance (equal loadings, intercepts and uniqueness) across gender. Therefore, the 9-item version of the RRS is a valid instrument for gender comparisons of Chilean adolescents.

**Table 4:** Measurement invariance for RRS model between male and female adolescents.

Model	χ^2^	df	D_SBS χ^2^	p_D_SBS χ^2^	RMSEA	CFI	TLI
0 Configural (without invariance)	100.326	52	–	–	0.063	0.918	0.886
1 Metric (loadings invariance)	102.490	59	1.509	0.982	0.056	0.926	0.910
2 Strong (loadings and intercepts)	109.659	66	7.199	0.408	0.053	0.926	0.919
3 Strict (loadings, intercepts and uniqueness)	120.146	75	6.318	0.707	0.051	0.923	0.926

χ*^2^, Chi square. df, Degree of freedom. SBS* χ*^2^, Satorra–Bentler Scaled Chi-square. D, Delta between a model and previous model. D_SBS* χ*^2^, Delta of Satorra–Bentler Scaled Chi-square. p_D_SBS* χ*^2^, Statistical significance of Delta of Satorra–Bentler Scaled of Chi-square. RMSEA, Root Mean Square Error of Approximations. CFI, Comparative Fit Index. TLI, Tuker–Lewis Index.*

### Testing Relationships With Structural Equation Model

Defined by the RRS model (without item 7) and verified by gender invariance, the fit of the structural model used to evaluate the relationships between the RRS subscales (DC and WF) and the Satisfaction with Life, Satisfaction with Food-related Life and Satisfaction with Family Life scales, as well as the relationships between the latter two and the SWLS, were tested.

**FIGURE 2:**
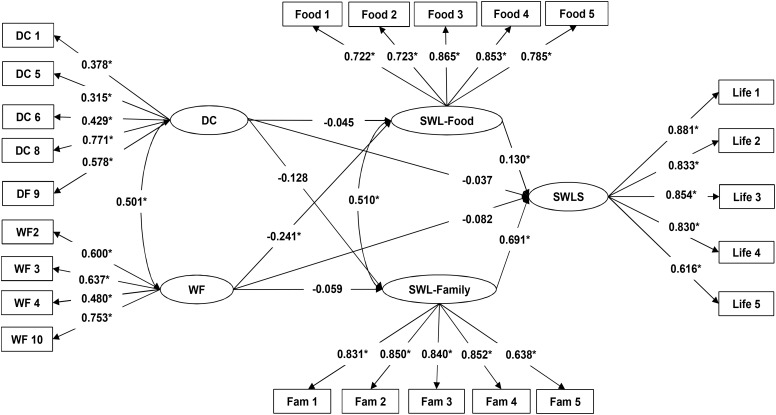
Path diagram of the model that explains the relationships between Dietary Concern (DC) and Weight Fluctuations (WF) and Satisfaction with Family Life (SWL-Family), Satisfaction with Food-related Life (SWL-Food), and Satisfaction with Life (SWLS), and between SWL-Food, SWL-Family and SWLS in the male adolescent sample. ^∗^*p* < 0.01. *Life 1. In most ways my life is close to my ideal*; *Life 2. The conditions of my life are excellent*; *Life 3. I am satisfied with my life*; *Life 4. So far I have gotten the important things I want in life*; *Life 5. If I could live my life over, I would change almost nothing.*; *Food 1. Food and meals are positive elements*; *Food 2. I am generally pleased with my food*; *Food 3. My life in relation to food and meals is close to ideal*; *Food 4. With regard to food, the conditions of my life are excellent*; *Food 5. Food and meals give me satisfaction in daily life.*; *Fam 1. In most ways my family life is close to my ideal*; *Fam 2. The conditions of my family life are excellent*; *Fam 3. I am satisfied with my family life*; *Fam 4. So far I have gotten the important things I want in family life*; *Fam 5. If I could live my family life over, I would change almost nothing.*; *DC 1. How often are you dieting?*; *DC 5. Would a weight fluctuation of 2.5 kilos affect the way you live your life?*; *DC 6 Do you eat sensibly in front of others and splurge alone?*; *DC 8. Do you have feelings of guilt after overeating?*; *DC 9. How conscious are you what you are eating?*; *WF 2. What is the maximum amount of weight (in kilos) you have ever lost within 1 month?*; *WF 3. What is the maximum amount of weight gain (in kilos) within a week?*; *WF 4. In a typical week, how much does your weight fluctuate?*; *WF 10. How many kilos over your desired weight were you at your maximum weight?*

Although the structural model for male adolescents had a significant Chi-square value (χ^2^ = 407.417, *p* < 0.01) (MacCallum et al., 2006), the rest of the fit indices indicated that the structural model showed an acceptable fit with the data (CFI = 0.928; TLI = 0.918; RMSEA = 0.055). The path coefficient between SWL-Food and SWLS was positive, confirming that food-related life satisfaction is positively related to overall life satisfaction in adolescents (H1). The path coefficient between SWL-Family and SWLS was also positive, supporting the finding that satisfaction with family life is positively related to overall life satisfaction in adolescents (H2) (**Figure 2**). The positive and significant correlation between SWL-Food and SWL-Family confirms that food-related life satisfaction is positively related to family life satisfaction in male adolescents (H3). The path coefficient between DC and SWLS was not significant, and thus did not support the hypothesis that dietary concern is negatively related to life satisfaction in male adolescents (H4). The path coefficient between WF and SWLS was not significant; this finding did not support the hypothesis that WF is negatively related to male adolescent life satisfaction (H4). Additionally, the non-significant path coefficient between DC and SWL-Food did not support the expectation that dietary concern is negatively related to satisfaction with food-related life in male adolescents (H6). However, the negative and significant path coefficient between WF and SWL-Food supported the hypothesis that WF is negatively related to satisfaction with food-related life in male adolescents (H7). The path coefficient between DC and SWL-Family was not significant and thus does not support the expectation that dietary concern is negatively related to satisfaction with family life in male adolescents (H8). The path coefficient between WF and SWL-Family also was not significant and therefore does not support the hypothesis that WF is negatively related to satisfaction with family life in male adolescents (H9).

**FIGURE 3:**
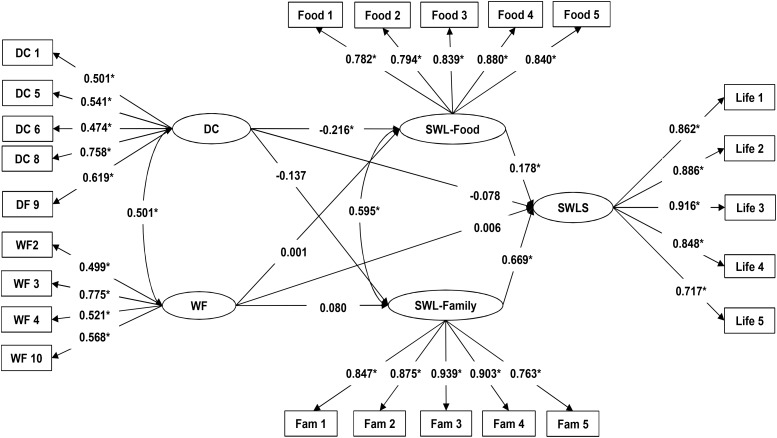
Path Diagram of model that explains the relationships between DC and WF and SWL-Family, SWL-Food and SWLS, and between SWL-Food, SWL-Family, and SWLS in the female adolescent sample. ^∗^*p* < 0.01.; *Life 1. In most ways my life is close to my ideal*; *Life 2. The conditions of my life are excellent*; *Life 3. I am satisfied with my life*; *Life 4. So far I have gotten the important things I want in life*; *Life 5. If I could live my life over, I would change almost nothing.*; *Food 1. Food and meals are positive elements*; *Food 2. I am generally pleased with my food*; *Food 3. My life in relation to food and meals is close to ideal*; *Food 4. With regard to food, the conditions of my life are excellent*; *Food 5. Food and meals give me satisfaction in daily life.*; *Fam 1. In most ways my family life is close to my ideal*; *Fam 2. The conditions of my family life are excellent*; *Fam 3. I am satisfied with my family life*; *Fam 4. So far I have gotten the important things I want in family life*; *Fam 5. If I could live my family life over, I would change almost nothing.*; *DC 1. How often are you dieting?*; *DC 5. Would a weight fluctuation of 2.5 kilos affect the way you live your life?*; *DC 6 Do you eat sensibly in front of others and splurge alone?*; *DC 8. Do you have feelings of guilt after overeating?*; *DC 9. How conscious are you what you are eating?*; *WF 2. What is the maximum amount of weight (in kilos) you have ever lost within 1 month?*; *WF 3. What is the maximum amount of weight gain (in kilos) within a week?*; *WF 4. In a typical week, how much does your weight fluctuate?*; *WF 10. How many kilos over your desired weight were you at your maximum weight?*

The SEM analysis for female adolescents also had a significant Chi-square value (χ^2^ = 4459.820, *p* < 0.01) (MacCallum et al., 2006), although the rest of the fit indices indicated that the structural model showed an acceptable fit with the data (CFI = 0.927; TLI = 0.917; RMSEA = 0.060). Similar to the results obtained for male adolescents, the path coefficient between SWL-Food and SWLS was positive, confirming that satisfaction with food-related life is positively related to overall life satisfaction in adolescents (H1). The path coefficient between SWL-Family and SWLS was positive, confirming that satisfaction with family life is positively related to overall life satisfaction in adolescents (H2) (**Figure 3**). The positive and significant correlation between SWL-Food and SWL-Family also confirms that food-related life satisfaction is positively related to family life satisfaction in female adolescents (H3). The path coefficient between DC and SWLS was not significant, and thus did not support the hypothesis that dietary concern is negatively related to life satisfaction in female adolescents (H4). The path coefficient between WF and SWLS also was not significant, which does not support the expectation that WF is negatively related to life satisfaction in female adolescents (H5). The negative and significant path coefficient between DC and SWL-Food supported the expectation that dietary concern is negatively related to satisfaction with food-related life in female adolescents (H6). However, the non-significant path coefficient between WF and SWL-Food did not support the hypothesis that WF is negatively related to satisfaction with food-related life in female adolescents (H7). The non-significant path coefficient between DC and SWL-Family did not support the expectation that dietary concern is negatively related to satisfaction with family life in female adolescents (H8). Likewise, the non-significant path coefficient between WF and SWL-Family did not support the hypothesis that WF is negatively related to satisfaction with family life in female adolescents (H9).

### The Moderating Role of Socioeconomic Status (SES)

Multi-group analyses that considered the SES as a categorical variable and compared the structural parameters for both SES conditions (High SES vs. Low SES) were made in each subsample. There were no significant statistical differences between “High SES” and “Low SES” for any regression parameter in the male subsample (results not shown, H10–H17).

**Table 5** shows the results for the subsample of female adolescents. There were significant statistical differences for the regression parameter of the relationship between food-related life satisfaction and life satisfaction (*p* = 0.001). Therefore, the SES variable was found to have a moderating role on this structural model parameter (H10), which presented a lower but non-statistically significant value (γ = −0.215, *P* = 0.057) for adolescents that belonged to the “High SES” group. In contrast, adolescents from the “Low SES” had a higher (γ = 0.342) and statistically significant value (*p* = 0.011). There were also significant statistical differences for the regression parameter of the relationship between WF and food-related life satisfaction (*p* = 0.015). Therefore, the SES variable was found to have a moderating role on this structural model parameter (H15), which presented a lower (γ = −0.423) and statistically significant value (*p* = 0.027) for adolescents who belonged to the “High SES” group. In contrast, adolescents from the “Low SES” had a higher (γ = 0.151), but non-statistically significant value (*p* = 0.127). There were no significant statistical differences between “High SES” and “Low SES” in the regression comparing the relationships between satisfaction with family life and life satisfaction, DC and life satisfaction, WF and life satisfaction, DC and food-related life satisfaction, DC and family life satisfaction and between WF and family life satisfaction in the female subsample. Therefore, SES was not found to have a moderating role on these structural model parameters (H11, H12, H13, H14, H16, and H17).

**Table 5:** Estimates for structural coefficients and moderation role of socioeconomic status in the model that explains the relationships between DC and WF and SWL-Family, SWL-Food and SWLS, and between SWL-Food, SWL-Family and SWLS in the female adolescent sample.

Structural path and direction			High SES		Low SES		*P*-value for estimate differences
			Estimate	*p*	Estimate	*p*	
SWL-Food	→	SWLS	-0.215	0.057	0.342	0.011	0.001^**^
SWL-Family	→	SWLS	0.925	0.000	0.538	0.000	0.121
DC	→	SWLS	-0.112	0.255	-0.007	0.918	0.350
WF	→	SWLS	-0.209	0.170	-0.015	0.819	0.258
DC	→	SWL-Food	0.064	0.713	-0.241	0.033	0.157
WF	→	SWL-Food	-0.423	0.027	0.151	0.127	0.015^*^
DC	→	SWL-Family	0.046	0.800	-0.140	0.205	0.385
WF	→	SWL-Family	-0.012	0.947	0.048	0.649	0.770

^∗^*p < 0.05. ^∗∗^p < 0.01.*

## Discussion

This is the first study to assess the joint relationship of the food and family domains with overall life satisfaction in adolescents of both genders. According to the “bottom–up” theoretical approach to life satisfaction (Brief et al., 1993) and the spillover model of domains interaction (Wu, 2009), the results of the SEM analyses confirm previous findings reported in undergraduate students (Schnettler et al., 2017c). Nevertheless, in the aforementioned study examining undergraduate students, the relationship between satisfaction with food-related life and life satisfaction was of low strength and the relationship between satisfaction with family life and life satisfaction was of medium strength. According to Cohen (1988), the results of the present study show that the relationship between satisfaction with food-related life and life satisfaction was of low strength and the relationship between satisfaction with family life and life satisfaction was of high strength. This finding is remarkable, as it shows that the family domain is of great importance to adolescent life satisfaction regardless of gender, despite the fact that adolescence is often associated with increasing autonomy (Pearson et al., 2017). This finding also suggests that the weight of each domain’s relation to well-being (Pavot and Diener, 1993) varies through distinct life stages. These findings should be confirmed by future research with samples including individuals in various stages of life, such as adolescents, emerging adults, adults and older adults.

Contrary to what was expected (Frasquilho et al., 2017; Gross-Manos, 2017), the multi-group analyses did not find evidence for a moderating role of SES on the relationship between satisfaction with family life and life satisfaction in subsamples of male and female adolescents. Therefore, in order to improve well-being, healthy relationships should be encouraged in families with adolescent children regardless of their gender and SES. In contrast, in the female subsample, SES was found to have a moderating role on the relationship between satisfaction with food-related life and life satisfaction. This result could be related to the fact that families from lower SES groups may not always have adequate access to food, both in term of quantity and quality (Lang and Barling, 2012). Indeed, lower levels of food-related life satisfaction may be related to unhealthier diets and skipped meals in undergraduate students from lower SES groups (Schnettler et al., 2015a,b). Hsieh (2016) stressed that domain importance plays a key role in the relationship between overall life satisfaction and domain satisfaction. Therefore, it is reasonable to hypothesize that individuals from lower SES groups are subject to food insecurity, which may increase the importance of the food domain and the strength of the relationship between food-related life satisfaction and overall life satisfaction. Regardless, although the food domain was less important than the family domain, it is possible to suggest that satisfaction in the food domain is important to improving the overall level of life satisfaction in adolescents of both genders, especially for female adolescents from lower SES groups.

Regarding dietary restrictions measured using the RRS, our results showed a better fit with the data as well as invariance across gender when 9 of the 10 original items of the scale were used. This result suggest that the RRS is gender- (Schnettler et al., 2014), culture- (van Strien et al., 2002; Mak and Lai, 2012; Meule, 2016) and age-sensitive, at least when RRS is used in samples of both genders. Therefore, studies that use the RRS to measure dietary restraint should assess its psychometric properties.

Contrary to what was expected, and considering that adolescents of both genders were sampled, our results confirm that dietary restraint is not related to life satisfaction across gender (Appleton and McGowan, 2006). This finding aligns with the results of a prior study, which also found no relationship between dietary restraint and life satisfaction in male undergraduate students (Orellana et al., 2016). However, further research is warranted, as our findings contradict other studies with mixed gender samples of undergraduate students which concluded that dietary restraint is associated with lower life satisfaction in females (Remick et al., 2009; Schnettler et al., 2017a) and in students of both genders (Schnettler et al., 2014).

Regardless, although dietary concern and WF were not related to overall life satisfaction in adolescents, these constructs were shown to be related to their satisfaction in the food domain, but in different ways. These findings are in line with studies that have reported that men are less concerned about their diet than women (Deliens et al., 2013) and are instead more focused on their body shape (Gempeler, 2006). One possible explanation may be associated with the prevalence of the traditional model of masculinity in Latin America (Toro-Alfonso et al., 2012) given that dietary concerns are seen as a sign of personal deficit and as “unmasculine” (Reas and Stedal, 2015; Yu et al., 2015). In this case, dietary concerns would not negatively affect their food-related life satisfaction (Orellana et al., 2016). Nevertheless, future research should compare male adolescents from Latin America to male adolescents from other cultures where the traditional model of masculinity is lower. In the female subsample, the negative association between dietary concern and food-related life satisfaction may be related to cultural pressure to comply with the ideal “thin body” that is commonly expected of women (Shomaker and Furman, 2009; Ferreiro et al., 2014; Suelter et al., 2016). Therefore, it is likely that the fear of gaining weight and not fitting the ideal female shape negatively affects satisfaction with food-related life in female adolescents.

Regarding a possible moderating role of SES, our results only supported the moderating role of this variable for the relationship between WF and food-related life satisfaction in the female subsample. One possible explanation for these results may be related to the practices used (or the absence of such practices) by mothers of different SES levels that are aimed at controlling the eating habits and weight of their adolescent children. Godoy et al. (2018) found that mothers from lower SES groups are more concerned that their adolescent children are fed than they are about nutritional quality, regardless of gender. In contrast, higher SES mothers control their daughters’ nutrition mainly by buying foods that are low in sugar and fat. In parallel, while mothers of lower SES groups do not pressure their children to lose weight or maintain a slender figure, regardless of gender, mothers from higher SES groups implement measures to control weight (dieting, restricting sugar consumption, physical exercise, among others) within their families, especially for their adolescent daughters. Although it was previously reported that adolescent girls experience greater interpersonal pressure to be thin than boys (Shomaker and Furman, 2009), our results suggest that interpersonal pressure to be thin may be greater in female adolescents of higher SES. Therefore, it is likely that WFs negatively and significantly affect food-related life satisfaction in female adolescents from higher SES groups, as being thin is given more importance in higher SES groups than in lower SES groups. Nevertheless, further research with samples of different compositions is needed to confirm these findings. Further research should also consider the role of the media and peer pressure on an adolescent’s desire to be thin (Shomaker and Furman, 2009; Suelter et al., 2016; Drouillet-Pinard et al., 2017) as it is possible that these types of pressures may be stronger in higher SES groups.

The lack of association between dietary concern and satisfaction with family life, and between WF and satisfaction with family life in both gender subsamples may be related to the fact that adolescent assessment of family life satisfaction does not consider food-related aspects, although food and family domains are positively related (Schnettler et al., 2017b,c).

One of the limitations of this study was its cross-sectional design and its use of a survey to obtain the data. In fact, all relationships were correlational, so causality cannot be determined. Therefore, new research is required to test causality and thereby must consider longitudinal, experimental or quasi-experimental designs. Another limitation of this study is related to the non-probabilistic nature of the sample and its relatively small size, as well as being conducted with adolescents from only one city in one country, which precludes the generalization of results. Also, all data were self-reported. Thus, responses may have been affected by social desirability. At the same time, we did not consider food-related parenting practices, family diet quality and the ways in which parents pressure their adolescent children to be thin and their influence on their children’s eating habits, weight and shape. In addition, we did not assess parent dietary restraint behavior or behavior modeling, which may influence their children’s behavior (Arroyo et al., 2017). All of these variables should be addressed in future studies.

In spite of these limitations, this study expands upon previous knowledge related to the positive relationships between life satisfaction, food-related satisfaction and family satisfaction, which suggests that these relationships are similar for both male and female adolescents. However, this study also provides new insights regarding these relationships, indicating the need to focus efforts on improving the levels of satisfaction with food-related life on more specific population groups, such as female adolescents from lower SES groups.

This is the first study to show that male and female adolescent concerns regarding weight and diet are negatively related to food-related life satisfaction in different ways, also showing that SES moderates the negative association between WF and food-related life satisfaction in female adolescents. Therefore, our results contribute by suggesting the need to develop interventions aimed at reducing DC in female adolescents and at reducing WFs in male adolescents and in female adolescents from higher socioeconomic backgrounds so that such interventions may improve food-related life satisfaction in adolescents who are restrained eaters. However, further research is required to identify a clinical cut-off point for these variables in order to identify which individuals need help, and to design effective interventions for those individuals. In addition, future research should test other variables that may moderate the relationship between food, family and life satisfaction and dietary restraint in adolescents, not only in Chile but also in other developing and developed countries.

## Ethics Statement

This study was conducted in accordance with the recommendations of the Ethics Committee of the Universidad de La Frontera (protocol number, 005/2016) and with the written informed consent of all subjects. All subjects gave written informed consent in accordance with the Declaration of Helsinki. The protocol was approved by Ethics Committee of the Universidad de La Frontera.

## Author Contributions

BS conceptualized and wrote the first manuscript draft, approved the statistical analysis, and the final version of the manuscript. EM-Z and KG guided the statistical analysis and made a critical analysis of the final version of the manuscript. GL and MD supervised data collection and made a critical analysis of the final version of the manuscript. CH prepared the literature review. All the authors read and approved the final manuscript.

## Conflict of Interest Statement

The authors declare that the research was conducted in the absence of any commercial or financial relationships that could be construed as a potential conflict of interest.

## Supplementary Material

DATA SHEET S1 Data of the scales and variables analyzed in the study with male and female adolescents; Restraint Scale (RRS), Diet Concern (DC), Weight Fluctuation (WF), Satisfaction with Life Scale (SWLS), Satisfaction with Food-related Life (SWL-Food) scale, Satisfaction with Family Life (SWL-Family) scale, socioeconomic status (SES), body mass index (BMI), family structure and number of family members.
